# Prevalence and determinants of antenatal tetanus vaccination in Sudan: a cross-sectional analysis of the Multiple Indicator Cluster Survey

**DOI:** 10.1186/s41182-022-00398-4

**Published:** 2022-01-10

**Authors:** Sagad Omer Obeid Mohamed, Esraa Mohammed Ahmed

**Affiliations:** Faculty of Medicine, University of Khartoum, Alqasr Avenue, P.O. Box 102, Khartoum, Sudan

**Keywords:** Antenatal care, ANC, Tetanus, Vaccine, Epidemiology, MICS

## Abstract

**Background:**

Tetanus vaccination is an indispensable component of the antenatal care (ANC) and is considered one of the most effective and protective measures against tetanus deaths. However, data on antenatal tetanus vaccination in Sudan are scarce. We aimed to explore the level of antenatal tetanus vaccination and to identify the influencing factors in a nationally representative population sample.

**Methods:**

We used the latest available data (2014) of the Sudan Multiple Indicator Cluster Survey (MICS), developed by the United Nations Children’s Fund (UNICEF). We assessed the level of antenatal tetanus vaccination among women of childbearing age who gave at least one birth preceding the survey and defined adequate antenatal tetanus vaccination according to the World Health Organization (WHO) recommendations. Data analysis was performed using descriptive statistics, bivariate analysis, and multivariate logistic regression analysis.

**Results:**

The total number of women of childbearing age involved in this analysis was 5433. Most of the participants (28.6%) were 25–29 years old, and vast majority of them (73.7%) live in rural areas. The prevalence of mothers who had adequate tetanus vaccination was 60.0%. Antenatal tetanus vaccination was significantly associated with higher level of mothers’ education (AOR = 1.70, 95% CI 1.25–2.32), higher household wealth index (AOR = 1.89, 95% CI 1.41–2.54), having four or more ANC visits (AOR = 1.49, 95% CI 1.30–1.71), and living in areas with low intensity of armed conflicts (AOR = 1.34, 95% CI 1.14–1.57).

**Conclusions:**

Socioeconomic status had a significant impact on adequate antenatal tetanus vaccination. The results indicate the existence of variable rates and unequal access to tetanus vaccination among women of childbearing age in Sudan.

## Introduction

Tetanus, a vaccine-preventable disease is caused by the anaerobic bacterium *Clostridium tetani*. Though it can affect all age groups, neonates and pregnant women remain at higher risk of infection, chiefly when deliveries occur under unsanitary conditions [1–3]. Neonatal tetanus is defined as tetanus occurring within the first 28 days of life, while maternal tetanus occurs during pregnancy or within the first 6 weeks after childbirth [1–3]. In developing countries, maternal and neonatal tetanus (MNT) is still a substantial cause of mortality and case fatalities from tetanus remain high [1]. Findings of Global Burden of Disease study conducted in 2015 revealed that there were 56,743 deaths due to tetanus and 19,937 of these deaths occurred in neonates. Of these neonatal tetanus deaths, 44% occurred in sub-Saharan Africa [3]. Moreover, neonatal tetanus tends to be under-reported as most childbirths in developing countries are home deliveries [1].

Tetanus toxoid is an effective and low-cost vaccine that could be safely given at any time during pregnancy to protect women of childbearing age and newborns [1, 4–6]. It has been estimated that antenatal tetanus vaccination can reduce neonatal mortality from tetanus by 94% [5]. For previously non-immunized adults or those with unknown immunization status, a total of five tetanus toxoid vaccine doses are recommended by the World Health Organization (WHO) to combat MNT [5–7]. Obtaining two doses of the tetanus toxoid vaccine is recommended during the first pregnancy. The first dose is provided at first contact with healthcare services, and the second dose is provided 4 weeks later and at least 2 weeks before delivery. While the third dose should be given at least 6 months after the second, the last two boosters can be provided during succeeding pregnancies or at least 1 year later [6, 7].

Several studies have examined the adequacy of antenatal tetanus vaccination in low-income countries and its associated factors. The studies have attributed it to several maternal-related influencing factors such as age, education, occupation, marital status, wealth status, birth order, antenatal care (ANC), media exposure, residence and distance from the healthcare facilities [8–12].

While Sudan is one of the 24 countries which are yet to eliminate MNT, the exact data on the incidence of neonatal tetanus and access to antenatal tetanus vaccination in Sudan are scarce [1]. In 1976, the expanded immunization program was first established in Sudan in a few states and eventually extended geographically to cover the whole country [13]. The country witnessed an improved infantile vaccination coverage rate, increasing from 62% for diphtheria–tetanus–pertussis in 2000 to 95% in 2017 [14]. Two studies conducted in 1990 found that tetanus infections caused more than one quarter (29%) of the neonatal mortality rate, ranging from 20 to 36 per 1000 live births. The studies also showed that neonatal deaths were significantly lowest when the tetanus vaccination was received during pregnancy and the umbilical cord was cleaned by hygienic methods [15, 16].

This study aimed to identify the extent of antenatal tetanus vaccination and their influencing factors in a nationally representative population sample. Understanding these factors can help design policies to reduce maternal and neonatal mortality in Sudan.

## Materials and methods

### Description of the dataset

This study was performed based on an analysis of the latest accessible data available from the Sudan Multiple Indicator Cluster Survey (MICS), a nationwide survey was conducted from August to December 2014 [17]. Sudan MICS is a part of the worldwide MICS program developed by United Nations Children's Fund (UNICEF) and the survey was funded by several international collaborators, including the WHO, World Food Program (WFP), and the United Nations Population Fund (UNFPA) [13]. The global MICS program aimed to collect internationally comparable data on several indicators of maternal and childhood health condition, allowing countries to generate conclusive evidence for usage in health policies, assessing the current situation, and monitoring progress toward the achievement of the millennium development goals and other agreed-upon commitments [17].

The dataset was described and used by previous studies [18–20]. As previously reported, the study used a stratified two-stage clustered sampling method to recruit the participants. The overall target sample size of the study was 18,000 households and the total number of households reached 17,142. Of these, 16,801 households were successfully interviewed by trained interviewers using a questionnaire consisting of demographic characteristics, reproductive history, antenatal and postnatal care, and family planning. For this study, we included women of childbearing age (15–49 years) who gave at least one birth preceding the survey, with complete data on tetanus vaccination status.

### Study population and variables

Adequate tetanus vaccination among women of childbearing age was defined previously as either receiving two doses of tetanus toxoid vaccine in the last pregnancy, more than one dose within the latest 3 years of birth, more than two doses within the latest 5 years of birth, more than three doses within the latest within 10 years of birth, or more than four doses at any time [6, 7]. Accordingly, the dependent variable (adequate tetanus vaccination) was coded as a binary (yes, no) for further analysis. The interviewers asked the participants to present their vaccination cards to record the dates of vaccination and to refer to information from the cards when available.

Based on the availability of the data collected, the study has following variables: age group (15–19, 20–24, 25–29, 30–34, 35–39, 40–44, 45–49 years); mother education (none, primary, secondary, higher); household wealth quintile index (poorest, second, middle, fourth, richest); ANC visits (less than four, four or more); and area of residence (urban, rural). In addition, as Sudan has a long history of armed conflicts associated with poor health situation, the country’s states according to the armed conflicts intensity. A previous study done by Dahab et al. created this parameter by using the scale of the Heidelberg Institute for International Conflict Research (HIIK) and they classified the armed conflicts intensity in South Kordofan, Blue Nile, and the five states of Darfur was classified as high [18].

### Data analysis

A descriptive statistics method was used to describe the variables and calculate the prevalence of adequate tetanus vaccination. We examined the relationship between adequate tetanus vaccination and the previously described factors. The variables that showed a statistically significant relationship at the bivariate analyses level (*p* < 0.05) were evaluated through multiple logistic regression analysis. The results were then described in odds ratios with the 95% confidence intervals. The results of all analyses with *p*-values < 0.05 were considered significant. Statistical analyses were performed using the SPSS software version 20 (SPSS Inc., Chicago, IL, USA).

## Results

Among the 18,302 women of childbearing age who were interviewed in the survey, 5622 women who gave at least one birth preceding the survey were the target of this analysis. However, the total number of women with complete data on tetanus vaccination status and involved in this analysis was 5433. Most of the participants (28.6%) were 25–29 years old, and vast majority of them (73.7%) live in rural areas. The findings of the analysis revealed that 80.9% of the mothers had at least one ANC visit, while 64.1% of them had at least four ANC visits. However, only 28.4% of women gave birth in a healthcare institution. The sociodemographic characteristics of the participants are presented in Table 1.

**Table 1:** Sociodemographic characteristics of the participants

Variable	Level	Total no.	Percentage (%)
Age group	15–19	379	7.0
	20–24	1099	20.2
	25–29	1554	28.6
	30–34	1154	21.2
	35–39	878	16.2
	40–44	286	5.3
	45–49	83	1.5
Area of residence	Rural	4001	73.7
	Urban	1432	26.3
Wealth index quintile	Poorest	1199	22.1
	Second	1192	21.9
	Middle	1143	21.0
	Fourth	1072	19.7
	Richest	827	15.2
Mother education	None	2153	39.6
	Primary	1958	36.0
	Secondary	919	16.9
	Higher	403	7.4
Armed conflict intensity	High intensity areas	1962	36.1
	Low-intensity areas	3471	63.9
Ever received ANC	Yes	4395	80.9
	No	1038	19.1
Number of ANC visits*	Less than four	1562	35.9
	Four or more	2784	64.1
Place of childbirth	Home delivery	3889	71.6
	Health institution delivery	1544	28.4
Adequate tetanus vaccination	No	2175	40.0
	Yes	3258	60.0

*Data of 1087 participants were missing

### Uptake of tetanus vaccine

Table 2 shows the regression analysis results of the predictors of antenatal tetanus vaccination among the participants. The finding of this analysis revealed that the participants’ education was significantly associated with adequate tetanus vaccination. The odds of having antenatal tetanus vaccination was 1.5 and 1.7 times higher among the participants with secondary and higher education compared to women from the none group [secondary (AOR = 1.51, 95% CI 1.22–1.86), higher (AOR = 1.70, 95% CI 1.25–2.32)]. In addition, the participants from higher household wealth index [fourth (AOR = 1.66, 95% CI 1.31–2.11), richest (AOR = 1.90, 95% CI 1.41–2.54)] had higher rates of antenatal tetanus vaccination compared to others.

**Table 2:** Regression results of adequate tetanus vaccination among the participants

Variable	Level	Adequate tetanus vaccination		
		(%)	COR	AOR *
Age group	15–19	61.2%	Ref	Ref
	20–24	64.4%	1.14 (0.90–1.46)	1.01 (0.76–1.35)
	25–29	62.2%	1.04 (0.83–1.31)	0.88 (0.67–1.16)
	30–34	59.2%	0.92 (0.72–1.17)	0.84 (0.63–1.12)
	35–39	53.9%	0.74 (0.58–0.95)	0.64 (0.48–0.87)
	40–44	52.3%	0.69 (0.51–0.95)	0.65 (0.45–0.95)
	45–49	54.2%	0.75 (0.46–1.21)	1.01 (0.56–1.83)
Area of residence	Rural	56.5%	Ref	Ref
	Urban	69.5%	1.75 (1.54–1.99)	1.01 (0.86–1.19)
Wealth index quintile	Poorest	45.0%	Ref	Ref
	Second	56.0%	1.55 (1.32–1.82)	1.26 (1.03–1.55)
	Middle	58.7%	1.74 (1.47–2.04)	1.31 (1.05–1.63)
	Fourth	69.8%	2.81 (2.36–3.34)	1.66 (1.31–2.11)
	Richest	76.3%	3.91 (3.24–4.76)	1.89 (1.41–2.54)
Mother education	None	48.0%	Ref	Ref
	Primary	63.5%	1.89 (1.66–2.14)	1.31 (1.12–1.54)
	Secondary	72.1%	2.81 (2.38–3.32)	1.51 (1.22–1.86)
	Higher	79.0%	4.08 (3.16–5.27)	1.70 (1.25–2.32)
Armed conflict intensity	High intensity areas	56.3%	Ref	Ref
	Low-intensity areas	62.0%	1.27 (1.13–1.42)	1.34 (1.14–1.57)
Number of ANC visits	Less than four	60.3%	Ref	Ref
	Four or more	72.2%	1.71 (1.50–1.95)	1.49 (1.30–1.71)

*Variables included into the model for the adjusted odds ratios: age group, mother education, wealth quintile index, ANC visits, area of residence, and the armed conflict intensity

Regarding the area of residence, no significant difference among the participants from rural and urban areas was observed. However, antenatal tetanus vaccination was significantly higher among women from areas with low intensity of armed conflicts compared to others (AOR = 1.34, 95% CI 1.14–1.57). In addition, having four or more ANC visits was another predictor of antenatal tetanus vaccination among the participants (AOR = 1.49, 95% CI 1.30–1.71).

## Discussion

It has been challenging to improve maternal and neonatal health in low-income countries, where the limited access to health services renders women vulnerable to worse health outcomes. Of these essential health services, antenatal tetanus vaccination among the participants remains a primary public health problem in developing countries. This study aimed to fill the current gap in the literature about antenatal tetanus vaccination in Sudan. Overall, the study results show that the proportion of pregnant women with antenatal tetanus vaccination was 60.0%. This was associated with the mothers’ education, household wealth index, adequacy of ANC visits, and residence in areas with low intensity of armed conflicts. However, other studies from other sub-Saharan African countries reported higher prevalence rates of receiving adequate antenatal tetanus immunization [8, 21–24]. For example, a cross-sectional survey of 8722 women in Sierra Leone in 2017 showed that the prevalence of antenatal tetanus vaccination reached 81.9% [22]. Also, it has been estimated that antenatal tetanus vaccination reached 89% and 91% in 2017 in Kenya and Zambia, respectively [24]. The noticeable high rate of adequate ANC visits in the Sierra Leonean study (85.5%) compared to our study could explain the differences in rates of antenatal tetanus vaccination. Also, the poorer economic status in Sudan, a low-income country, compared to Kenya and Zambia, which are lower-middle-income countries, and the long history of civil war in Sudan could explain the lower rate of vaccination in our study [18, 25].

The study results show that both individual- and community-level factors were associated with antenatal tetanus vaccination. The finding that maternal education has a significant relationship with antenatal tetanus vaccination is consistent with the results of an analysis of the Ethiopian Demographic and Health Survey 2016, reporting that the odds of having a protected birth against tetanus was 1.36 times higher among mothers with secondary and above educational level than those without formal education [8]. Hence, it would be safe to say that education can improve the uptake of tetanus vaccination as educated women tend to have a greater decision-making power regarding their health and have the freedom to travel outside the home to seek healthcare.

Our findings revealed that antenatal tetanus vaccination was significantly associated with a higher number of ANC visits, consistent with other studies done in Ethiopia and Sierra Leone [8, 18]. The ANC visits offer opportunities to reach pregnant women with information on several health practices and interventions that may be vital to their health and well-being. Women who attend more ANC visits are more likely to be informed about the importance of antenatal vaccination than those who attend less [8, 22].

The sociocultural differences and inequality in the distribution of healthcare facilities could contribute to the observed regional variation of antenatal tetanus vaccination, a similar finding reported by other studies conducted in several developing countries [8, 21–23]. However, the armed conflicts in the case of Sudan put further challenge, since these high-risk areas are often geographically remote with poor infrastructure and health system. Inequalities in access to healthcare could threaten the overall growth development of a country.

We found that antenatal tetanus vaccination tends to be higher among the younger age groups compared to the others. However, the findings of previous studies were controversial [12, 26]. A cross-sectional survey of 9583 women in Ivory Coast conducted by Yaya et al. showed that women in higher age groups have higher rates of antenatal tetanus vaccination but they suggested that this relationship was affected by other factors such as the exposure to multiple pregnancies among older women and attending more ANC visits [23]. On the other hand, other factors could be the reasons for the higher vaccination rate among younger women in this study such as the recent improved access to modern media sources and the formal female education.

Similar to our findings, studies identified wealth status as one of the determinants of antenatal tetanus vaccination [9]. Even with the affordability of the vaccines and being free of charge, the accessibility to healthcare centers could be the cause of differences in rates of antenatal tetanus vaccination. The financial means enable women from wealthy households to get easier access to healthcare services compared to those from poor households [23].

As an implication of this study, the present data could permit targeted tracking of the vulnerable subgroups to improve ANC and tetanus vaccination. For example, a previous successful scale-up program was established in Darfur, Sudan, from 2007 to 2009 [27]. The interpersonal communication and mass education campaigns increased coverage and access to ANC health services among 15- to 49-year-old women. The number of women who received ANC visits and tetanus vaccination had risen significantly in the follow-up than in the initial survey [27].

Though this study aimed to quantify the problem in a nationally representative population sample, it still has some limitations. The first important limitation of the study is that the self-reported nature of the study raises the possibility of recall bias. The second limitation of the study is that the secondary data source used for this analysis allowed us limited control over the correlated variables to include in the analysis.

## Conclusions

Having antenatal tetanus vaccination was significantly associated with individual and community-level factors. Household wealth and mothers’ education have a significant impact on rates of tetanus vaccination in Sudan. In addition, the findings highlight the unequal rates of antenatal tetanus vaccination among women of childbearing age in Sudan. Interventions to improve the use of maternal health services and to reduce the observed inequalities between states should include the promotion of female education and improving the affordability of tetanus vaccine across different regions of the country.

## Data Availability

The dataset used during the current study is available from the corresponding author on reasonable request. The underlying dataset that was further analyzed in this study is available from https://mics.unicef.org/surveys.

## Abbreviations

TTCV: Tetanus toxoid-containing vaccine
MNT: Maternal and neonatal tetanus
UNICEF: United Nation Children Fund
MICS: Multiple Indicator Cluster Survey
WHO: World Health Organization
UNFPA: United Nations Population Fund

## References

[1] Thwaites CL, Beeching NJ, Newton CR (2015). Maternal and neonatal tetanus. Lancet.

[2] Demicheli V, Barale A, Rivetti A (2015). Vaccines for women for preventing neonatal tetanus. Cochrane Database Syst Rev.

[3] Kyu HH, Mumford JE, Stanaway JD (2017). Mortality from tetanus between 1990 and 2015: findings from the global burden of disease study 2015. BMC Public Health.

[4] Ridpath AD, Scobie HM, Shibeshi ME (2017). Progress towards achieving and maintaining maternal and neonatal tetanus elimination in the African region. Pan Afr Med J.

[5] Blencowe H, Lawn J, Vandelaer J, Roper M (2012). Tetanus toxoid immunization to reduce mortality from neonatal tetanus. Int J Epidemiol.

[6] World Health Organization (WHO). Maternal and neonatal tetanus elimination. https://www.who.int/reproductivehealth/publications/maternal_perinatal_health/immunization_tetanus.pdf Accessed 15 Jun 2021.

[7] Sherley J, Newton S (2020). The association between area of residence and sufficient antenatal tetanus vaccination in women ages 15–49 in Afghanistan: an analysis of the 2015 DHS dataset. Global Health Res Policy.

[8] Teshale AB, Tesema GA (2020). Determinants of births protected against neonatal tetanus in Ethiopia: a multilevel analysis using EDHS 2016 data. PLoS ONE.

[9] Haile ZT, Chertok IRA, Teweldeberhan AK (2013). Determinants of utilization of sufficient tetanus toxoid immunization during pregnancy: evidence from the Kenya demographic and health survey, 2008–2009. J Commun Health.

[10] Anatea MD, Mekonnen TH, Dachew BA (2018). Determinants and perceptions of the utilization of tetanus toxoid immunization among reproductive-age women in Dukem Town, Eastern Ethiopia: a community-based cross-sectional study. BMC Int Health Hum Rights.

[11] Hassan AM, Shoman AE, Abo-Elezz NF, Amer MM (2016). Tetanus vaccination status and its associated factors among women attending a primary healthcare center in Cairo governorate, Egypt. J Egypt Public Health Assoc.

[12] Dubale Mamoro M, Kelbiso HL (2018). Tetanus toxoid immunization status and associated factors among mothers in Damboya Woreda, Kembata Tembaro Zone, SNNP, Ethiopia. J Nutr Metab.

[13] Ali AHM, Abdullah MA, Saad FM, Mohamed HAA (2020). Immunisation of children under 5 years: mothers’ knowledge, attitude and practice in Alseir locality, Northern State, Sudan. Sudan J Paed.

[14] Ahmed N, DeRoeck D, Sadr-Azodi N (2019). Private sector engagement and contributions to immunisation service delivery and coverage in Sudan. BMJ Glob Health.

[15] Taha TE, Gray RH, Abdelwahab MM (1993). Determinants of neonatal mortality in central Sudan. Ann Trop Paediatr.

[16] Mustafa BE, Omer MI, Aziz MI, Karrar ZE (1996). Neonatal tetanus in rural and displaced communities in the East Nile Province. J Trop Pediatr.

[17] Surveys—UNICEF MICS. Mics.unicef.org. https://www.unicef.org/sudan/media/1146/file/Multiple-Indicator-Cluster-Survey-Report-2014%20.pdf. Accessed 15 Jun 2021.

[18] Dahab R, Bécares L, Brown M (2020). Armed conflict as a determinant of children malnourishment: a cross-sectional study in The Sudan. BMC Public Health.

[19] Mohamed SOO, Alawad MOA, Ahmed AAM (2020). Access to oral rehydration solution and zinc supplementation for treatment of childhood diarrhoeal diseases in Sudan. BMC Res Notes.

[20] Mohamed SOO, Ali HMA, Ali EAM, Mustafa SAM, Hassan SHM (2020). Knowledge, attitude, and testing of human immunodeficiency virus infection among 15- to 49-year-old women in Sudan: an analysis of the united nations children’s fund-multiple indicator cluster survey. Dr Sulaiman Al Habib Med J.

[21] Haile ZT, Chertok IRA, Teweldeberhan AK (2013). Determinants of utilization of sufficient tetanus toxoid immunization during pregnancy: evidence from the Kenya demographic and health survey, 2008–2009. J Commun Health.

[22] Yaya S, Kota K, Buh A, Bishwajit G (2020). Prevalence and predictors of taking tetanus toxoid vaccine in pregnancy: a cross-sectional study of 8,722 women in Sierra Leone. BMC Public Health.

[23] Yaya S, Kota K, Buh A, Bishwajit G (2019). Antenatal visits are positively associated with uptake of tetanus toxoid and intermittent preventive treatment in pregnancy in Ivory Coast. BMC Public Health.

[24] Tikmani SS, Ali SA, Saleem S (2019). Trends of antenatal care during pregnancy in low- and middle-income countries: findings from the global network maternal and newborn health registry. Semin Perinatol.

[25] World bank country and lending groups—world bank data help desk [Internet]. Worldbank.org. Available from: https://datahelpdesk.worldbank.org/knowledgebase/articles/906519-world-bank-country-and-lending-groups accessed 2021 Nov 24.

[26] Singh A, Pallikadavath S, Ogollah R, Stones W (2012). Maternal tetanus toxoid vaccination and neonatal mortality in rural north India. PLoS ONE.

[27] Adam IF, Nakamura K, Kizuki M, Al Rifai R, Vanching U (2015). Relationship between implementing interpersonal communication and mass education campaigns in emergency settings and use of reproductive healthcare services: evidence from Darfur, Sudan. BMJ Open.

